# Biomimetic Implant Surface Functionalization with Liquid L-PRF Products: In Vitro Study

**DOI:** 10.1155/2018/9031435

**Published:** 2018-05-08

**Authors:** Marco Lollobrigida, Manuela Maritato, Giuseppina Bozzuto, Giuseppe Formisano, Agnese Molinari, Alberto De Biase

**Affiliations:** ^1^Department of Oral and Maxillofacial Sciences, Sapienza University of Rome, Rome, Italy; ^2^Private Practice, Rome, Italy; ^3^National Centre of Drug Research and Evaluation, Istituto Superiore di Sanità, Rome, Italy

## Abstract

**Objective:**

Platelet-rich fibrin (PRF) clots and membranes are autologous blood concentrates widely used in oral surgical procedures; less is known, however, about the liquid formulations of such products. The aim of this in vitro study is to assess the behavior of different implant surfaces when in contact with two liquid leucocyte- and platelet-rich fibrin (L-PRF) products.

**Methods:**

Six commercial pure titanium discs, of 9.5 mm diameter and 1.5 mm thickness, were used. Three of these samples had a micro/nano-rough surface; three were machined. Three different protocols were tested. Protocols involved the immersion of the samples in (1) a platelets, lymphocytes, and fibrinogen liquid concentrate (PLyF) for 10 minutes, (2) an exudate obtained from L-PRF clots rich in fibronectin and vitronectin for 5 minutes, and (3) the fibronectin/vitronectin exudate for 2 minutes followed by immersion in the PLyF concentrate for further 8 minutes. After these treatments, the samples were fixed and observed using a scanning electron microscope (SEM).

**Results:**

Under microscopic observation, (1) the samples treated with the PLyF concentrate revealed a dense fibrin network in direct contact with the implant surface and a significant number of formed elements of blood; (2) in the samples treated with the fibronectin/vitronectin exudates, only a small number of white and red blood cells were detectable; and (3) in samples exposed to the combined treatment, there was an apparent increase in the thickness of the fibrin layer. When compared to the machined surface, the micro/nano-rough samples showed an overall increased retention of fibrin, leading to a thicker coating.

**Conclusions:**

Liquid L-PRF products promote the formation of a dense fibrin clot on micro/nano-rough implant surfaces in vitro. The adjunctive treatment of surfaces with the fibronectin/vitronectin exudate could provide support to contact of the fibrin with the surface, though it is not essential for the clot formation. Further studies are necessary to better elucidate the properties and benefits of liquid L-PRF products.

## 1. Introduction

Implant supported oral rehabilitation has increasingly improved the treatment options for edentulous patients, reporting high long-term survival and success rates [1, 2]. However some clinical conditions can affect osseointegration, significantly reducing the success rate of dental implants. An increased rate of implant loss has been reported in irradiated patients [3], in patients receiving bisphosphonates [4], and in individuals with severe periodontal disease [5]. In these patients, implant failures can occur at an early stage of peri-implant bone healing, thus suggesting a role for local factors. One fundamental phase of the healing process is the formation of a stable fibrin clot in contact with the implant surface to provide a provisional scaffold for the migration of differentiating osteogenic cells towards the implant surface [6]. This has led to the development of surfaces with specific micro- and nanotopographies and biomimetic characteristics to promote fibrin adhesion and improve osseointegration. If, on the one hand, dental implants with surface microtopography have become a standard of care, on the other very few brands commercially offer micro-nano-textured surfaces, whilst biomimetic approaches for implant functionalization are still not available for clinical use. These treatments generally involve immobilization of specific peptides on the implant surface during the production process. Another possible approach involves functionalizing the implant surface with the patient's autologous blood immediately before placement.

Today, platelet concentrates include several biologic products, commonly referred to as platelet-rich plasma (PRP) and platelet-rich fibrin (PRF), used to facilitate and promote wound healing. Specific formulations also include leucocyte- and platelet-rich fibrin (L-PRF) products [7]. Platelet concentrates are obtained through the centrifugation of a whole blood sample, discarding red blood cells and concentrating the components of use for therapeutic purposes, that is, fibrinogen, fibrin, platelets, growth factors, leukocytes, and other circulating cells [8, 9]. By pressing the fibrin clots obtained through the centrifugation, an exudate is formed which is rich in growth factors and serum proteins, including fibronectin and vitronectin [10]. These play an important role in cell adhesion and migration into the fibrin clot [11]. Liquid concentrates rich in platelets, lymphocytes, plasma proteins, and fibrinogen can also be obtained by shorter blood centrifugation. This plasma fibrinogen concentrate can be collected before coagulation and used for local delivery of growth factors similarly to the clots. Compared to PRF clots however, less is known as to the properties and the potential applications of liquid PRF products.

Although platelet concentrates are widely used in bone regeneration procedures, their role in relation to implant osseointegration remains poorly investigated. The purpose of this in vitro study was thus to evaluate the effects of treating rough and smooth implant surfaces with two liquid leucocyte- and platelet-rich fibrin (L-PRF) products.

## 2. Materials and Methods

### 2.1. Liquid Platelet Concentrate and Exudate Preparation

**Figure 1:**
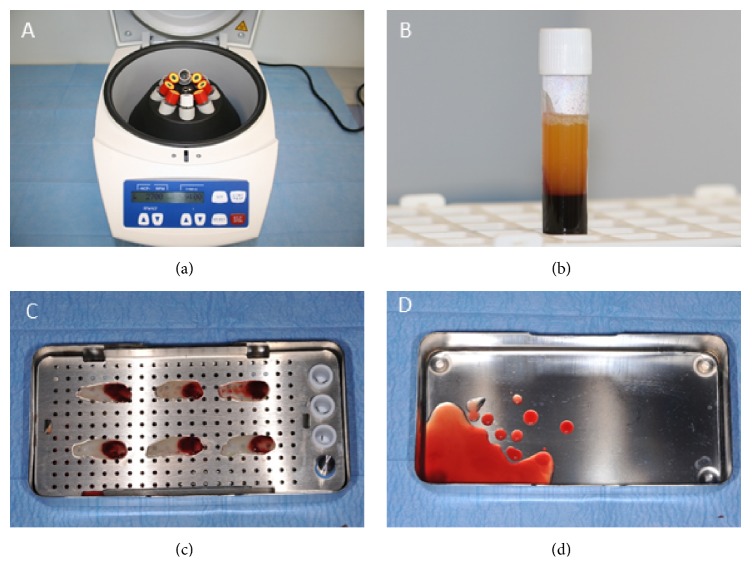
L-PRF products preparation. (a) The centrifuge used for the study with the vacutainer tubes in place. (b) PLyF concentrate on the top of the white vacutainer. (c) L-PRF membranes obtained after compression of the clots. (d) Exudate rich in fibronectin and vitronectin derived from membranes compression.

Blood samples (9 cc each) were collected in 6 red cap vacuum tubes (IntraSpin™, Intra-Lock International, Boca Raton, FL) to obtain the L-PRF clots and two white cap vacuum tubes (IntraSpin, Intra-Lock International, Boca Raton, FL) to produce the liquid concentrate. The total of 8 test tubes was then placed within the centrifuge (IntraSpin, Intra-Lock International, Boca Raton, FL) at opposing positions to balance the rotor (Figure 1(a)). After 3 minutes of centrifugation at 2700 rpm, the process was stopped and the 2 white tubes were removed, and the centrifuge restarted for a remaining period of 9 minutes. 3 cc of liquid (PLyF concentrate) was taken from the top of test tubes with the white caps (Figure 1(b)). After a total of 12 (3 + 9) minutes of centrifugation at 2700 rpm, the L-PRF clots were removed from the test tubes. The red layer containing the red blood cells was gently separated using a sterile instrument. The clots were then placed on a sterile metal grid and compressed under the weight of a sterile metal plate (Figure 1(c)), without applying any manual pressure (Xpression™ Kit, Intra-Lock International, Boca Raton, FL). After 5 minutes the L-PRF membranes were formed and the expressed exudate rich in fibronectin and vitronectin was collected at the base of the metal box (Figure 1(d)).

### 2.2. Treatment of Titanium Discs

Twelve sterile commercial pure titanium discs (ASTM Grade 4) of 9.5 mm diameter and 1.5 mm thickness were used for this study. Six discs had a rough fractal nanosurface (Ossean® surface, Intra-Lock International, Boca Raton, FL, USA) and six a machined surface. The discs were then divided into three separate groups using a multiwell cell culture plate: four discs, two machined and two rough (P_R_ and P_L_), were immersed in the PLyF concentrate for 10 minutes; four discs, two machined and two rough (E_R_ and E_L_), were immersed in the fibronectin and vitronectin exudate for 5 minutes; four discs, two machined and two rough (EP_R_ and EP_L_), were first immersed in the fibronectin and vitronectin exudate for 2 minutes and then in the PLyF liquid for 8 minutes. The samples were then fixed with 2% glutaraldehyde in 0.1 M cacodylate buffer (pH 7.4) and successively analyzed using a field emission gun scanning electron microscope (FEG-SEM) (Inspect FTM, FEI Company, Hillsboro, OR, USA) at different magnifications and an acceleration voltage of 10 kV.

## 3. Results

**Figure 2:**
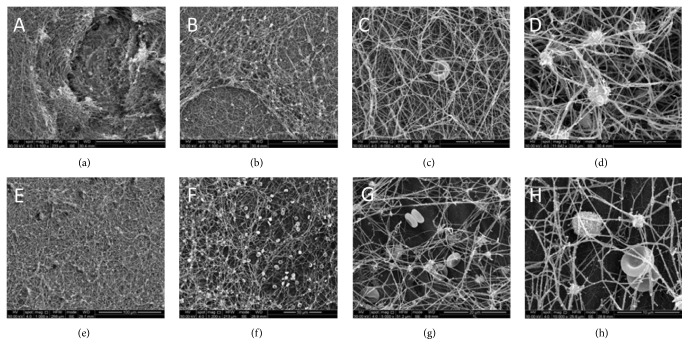
SEM images of titanium samples after immersion in the PLyF concentrate for 10 minutes. (a–d) Samples with rough surface (P_R_). (e–h) Samples with machined surface (P_L_). A dense fibrin network has formed on the surfaces with abundant thrombocytes, erythrocytes, and leukocytes.

On microscopic analysis, the P_R_ samples revealed a high-density, small-meshed fibrin network (Figures 2(a)–2(d)). A significant number of red blood cells, white blood cells, and platelets were also observed, both in contact with the disc surface and within the fibrin network. The fibrin clot was of variable thickness and direct contact between the fibrin network and the implant surface could be observed. Partial detachment of the fibrin from the titanium surface was also noted in some areas of the sample.

In samples P_L_ a wide-meshed fibrin network was observed (Figures 2(e)–2(h)). As compared to P_R_, the fibrin layer had a reduced thickness with few contact points between the fibrin and the implant surface. Similarly to P_R_ samples, a number of blood cells and platelets were trapped in the fibrin network.

**Figure 3:**
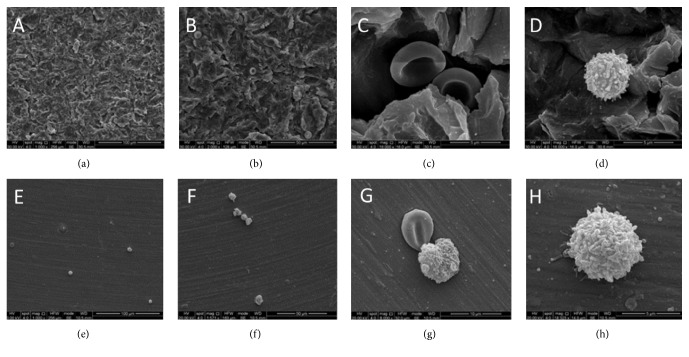
SEM images of titanium samples after immersion in the fibronectin and vitronectin exudate for 5 minutes. (a–d) Samples with rough surface (E_R_). (e–h) Samples with machined surface (E_L_). Scarce erythrocytes and leukocytes can be clearly detected on both the surfaces.

In samples E_R_ (Figures 3(a)–3(d)) and E_L_ (Figures 3(e)–3(h)) no significant biologic process could be observed. Few white and red blood cells could be identified in contact with surface irregularities.

**Figure 4:**
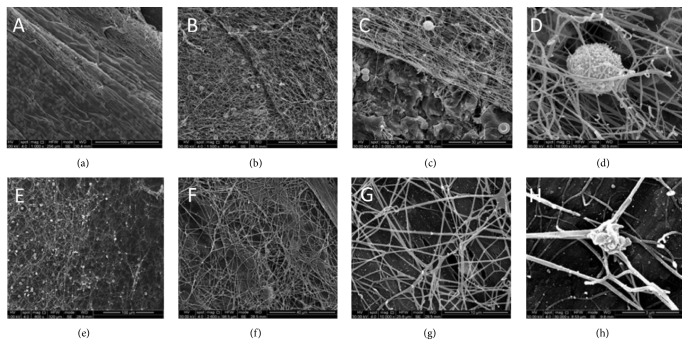
SEM images of titanium samples after immersion in the fibronectin and vitronectin exudate for 2 minutes and then in the PLyF concentrate for further 8 minutes. (a–d) Samples with rough surface (EP_R_). (e–h) Samples with machined surface (EP_L_). Compared to P_R_ and P_L_ samples, a major quantity of fibrin has formed on the surfaces.

Similarly to what was observed in the P_R_ samples, a dense, small-meshed fibrin layer had formed on the EP_R_ discs (Figures 4(a)–4(d)). Compared to P_R_, a greater quantity of fibrin had formed and a relatively higher number of formed blood elements were also detected within the fibrin clot. Several areas showed partial or total detachment of the fibrin layer from the surface, probably occurring during the fixation process.

Finally, the EP_L_ samples were covered with a wide-meshed fibrin network with several formed blood elements (Figures 4(e)–4(h)). Compared to EP_R_, the fibrin layer on EP_L_ showed a reduced thickness in all the observed areas. Similarly to P_L_, few direct contacts between the fibrin network and the implant surface were identified.

## 4. Discussion

The results of this study have shown that the contact of a micro/nano-rough implant surface with a liquid blood concentrate allows formation of a stable fibrin layer containing platelets and leucocytes. SEM micrographs have also suggested that fibrin clot formation may be further supported by adjunctive pretreatment of samples with an exudate containing fibronectin and vitronectin.

Platelet concentrates have been used widely in several branches of medicine to improve repair of soft tissue. In recent years, particular attention has been given to the potential for interaction between platelet concentrates and bone healing. In an experimental rat model of femoral fracture, Dülgeroglu and Metineren [12] observed increased bone formation after 28 days of healing when PRF clots were applied to bone. Similarly, Nagata et al. [13] observed significantly increased bone formation in surgically created bone defects in rat calvaria treated with a combination of autogenous bone graft and platelet-rich plasma. In 2003, Schlegel et al. [14] tested the potential of PRP in artificial peri-implant bone defects in a dog model without conclusive results. Most recent research has focused on the use of PRF to enhance bone regeneration and osseointegration. PRF has proven effective in bone regeneration of peri-implant defects in both animal [15, 16] and human clinical studies [17, 18]. In a histometric study in rabbits, Öncü et al. [19] evaluated the effect on osseointegration of placing L-PRF within implant beds and implant prewetting in L-PRF clots before placement. The authors report increases in the rate and the amount of new bone formation in the experimental group compared to the control, especially in the early healing stages. In a histologic study in dogs, Neiva et al. [20] evaluated the effects of L-PRF around immediately placed implants with two different surfaces, micro/nano-rough surface (Ossean™ surface) and dual-etched. The authors reported a significantly increased bone formation by combining Ossean surface with L-PRF concluding that micro/nano-rough surface and L-PRF have a synergistic effect on peri-implant bone healing.

In the present study a different approach has been developed, consisting in soaking the titanium samples in a liquid platelet concentrate obtained after 3 minutes of centrifugation. The theoretical advantage of liquid concentrates instead of PRF clots is that fibrin polymerization occurs in direct contact with the implant surface. As demonstrated by the SEM images, fibrin can establish numerous contacts with the implant surface, providing a biologic coating. Although various efforts are being made to improve osseointegration by mechanisms such as implant surface coating, the most important biological event is the fibrin clot formation around the implant. The quality and stability of the fibrin clot are, in fact, a prerequisite for mesenchymal stem cell migration and differentiation into the osteoblast lineage and subsequent contact osteogenesis. From this perspective, L-PRF concentrates provide all the key agents necessary for the early stages of osseointegration: fibrin, platelets (and related growth factors), and leukocytes.

Bone healing is also influenced by a number of molecules including fibronectin and vitronectin. During the early healing phase, fibronectin is incorporated into the fibrin matrix affecting distinct platelet functions (adhesion aggregation, activation), as well as cell migration into the forming provisional matrix [21, 22]. Similarly, once incorporated into the fibrin clot vitronectin supports platelet adhesion and aggregation and, at later stages, contributes to cell adhesion to the extracellular matrix [23]. Based on this, some authors have proposed fibronectin and vitronectin-coated implants to enhance osseointegration [24–26]; despite some positive results obtained in vitro, however, the complexity and cost of such surface treatments have limited their widespread use. In this study, a simpler approach, consisting in soaking the surfaces in the L-PRF-derived products, has been adopted. In the case of the fibronectin/vitronectin exudate, protein adsorption occurs as a result of van der Waals forces or electrostatic interactions similarly to what happens in vivo. Moreover, in adjunction to the autologous origin of adsorbed molecules, the advantage of this procedure is that it is applicable during surgery [27].

The use of PRF preparations for the biomimetic coating of dental implants can promote peri-implant bone healing also through the local delivery of growth factors and proteins [28]. Various growth factors, including platelet-derived growth factor (PDGF) and transforming growth factor beta (TGF-*β*), are secreted by local platelet degranulation. Growth factors act as modulators of cellular activity, inducing specific responses in all phases of bone repair [29] and promoting angiogenesis [30]. From this perspective, PRF products may have an osteopromotive effect during peri-implant bone healing when associated with a nanotextured surface [31, 32]. Indeed, the specific L-PRF formulation used in this study could further support the healing process, participating in the initial inflammatory response. However the role of leukocytes contained in PRF still has to be elucidated.

Considering these properties, PRF products may find application in patients with impaired bone healing capacity, for example, those who have undergone radiation therapy. In these patients, osseointegration is negatively affected as a result of the hypocellular, hypovascular, and hypoxic tissue environment [33], with an increased risk of failure particularly in the maxilla and in grafted sites [34]. These products could also prove beneficial in immediate implant placement after extraction, to stimulate fibrin clot formation in the gap between the alveolar bone and the implant surface, in guided bone regeneration procedures with simultaneous implant placement [35], and in the regeneration of peri-implant defects [36]. However, rather than as hitherto described in the literature, this study proposes use of liquid L-PRF instead of clots in order to enhance direct contact of the fibrin layer with the implant surface, obtaining an immediate biofunctionalization of the surface.

## 5. Conclusion

This study represents a preliminary evaluation of surface treatment with liquid L-PRF products, with the main limitations being a small number of samples and the qualitative nature of the observations. The study results indicate that treatment of implant surfaces with liquid PRF leads to the formation of a stable and dense fibrin layer in direct contact with the implant surface, thus providing a biomimetic autologous coating. Compared to machined surfaces, the micro/nano-rough samples were found to be more retentive, leading to thicker coatings. Adjunctive treatment with the L-PRF clot exudate containing fibronectin and vitronectin seems to promote greater fibrin adhesion and formation when in combination with the liquid platelet concentrate. There is a need for additional studies to better elucidate the potential benefits of liquid PRF in enhancing osseointegration, particularly in those patients for whom implant therapies still encounter increased risk of failure.

## Data Availability

All data analyzed during this study are available from the corresponding author on reasonable request.
